# A male mouse model of WIN 55,212–2 self-administration to study cannabinoid addiction

**DOI:** 10.3389/fphar.2023.1143365

**Published:** 2023-03-27

**Authors:** María del Mar Cajiao-Manrique, Rafael Maldonado, Elena Martín-García

**Affiliations:** ^1^ Laboratory of Neuropharmacology-Neurophar, Department of Medicine and Life Sciences, Universitat Pompeu Fabra (UPF), Barcelona, Spain; ^2^ Hospital del Mar Medical Research Institute (IMIM), Barcelona, Spain; ^3^ Departament de Psicobiologia i Metodologia de les Ciències de la Salut, Universitat Autònoma de Barcelona (UAB), Barcelona, Spain

**Keywords:** cannabinoid addiction, mouse model, WIN 55,212-2 self-administration, persistence of response, motivation, compulsive-like behavior

## Abstract

We have established for the first time a mouse model of cannabinoid addiction using WIN 55,212–2 intravenous self-administration (0.0125 mg/kg/infusion) in C57Bl/6J mice. This model allows to evaluate the addiction criteria by grouping them into 1) persistence of response during a period of non-availability of the drug, 2) motivation for WIN 55,212–2 with a progressive ratio, and 3) compulsivity when the reward is associated with a punishment such as an electric foot-shock, in agreement with the Diagnostic and Statistical Manual of Mental Disorders 5th edition (DSM-5). This model also allows to measure two parameters that have been related with the DSM-5 diagnostic criteria of craving, resistance to extinction and reinstatement, and two phenotypic traits suggested as predisposing factors, impulsivity and sensitivity to reward. We found that 35.6% of mice developed the criteria of cannabinoid addiction, allowing to differentiate between resilient and vulnerable mice. Therefore, we have established a novel and reliable model to study the neurobiological correlates underlying the resilience or vulnerability to develop cannabinoid addiction. This model included the chemogenetic inhibition of neuronal activity in the medial prefrontal cortex to the nucleus accumbens pathway to assess the neurobiological substrate of cannabinoid addiction. This model will shed light on the neurobiological substrate underlying cannabinoid addiction.

## 1 Introduction

*Cannabis sativa* derivatives are the most used illicit drugs worldwide, with an increased consumption over the recent years. In Europe, cannabis use has enhanced from 5.7% in 2015 to 7.7% in 2022 in adults aged from 15 to 64 (European Monitoring Centre for Drugs and Drug Addiction, 2015; European Monitoring Centre for Drugs and Drug Addiction, 2022). However, attitudes toward cannabis use have softened since there is a growing social perception that cannabis is relatively harmless (Weiss and Volkow, 2022). This lack of risk perception has led to an increase in the prevalence of cannabis use disorder, previously defined as cannabis dependence (Zehra et al., 2018)*.*

Cannabis use disorder is defined as a chronically relapsing neuropsychiatric disorder diagnosed by applying the criteria defined in the 5th edition of the *Diagnostic and Statistical Manual of Mental Disorders* (DSM-5). In this edition, the term addiction is synonymous with severe substance-use disorder and requires the accomplishment of six out of eleven diagnostic criteria (Koob and Volkow, 2016).

Cannabis addiction results from the interaction between gene networks, epigenetics, and socio-environmental factors (Piazza and Deroche-Gamonet, 2013; Hamilton and Nestler, 2019; Lüscher et al., 2020; Maldonado et al., 2021). Not all individuals repeatedly exposed to the drug make this transition to addiction (Piazza and Deroche-Gamonet, 2013), raising the question of why some vulnerable individuals become addicted while others are resilient. Although multiple neuroadaptations induced by cannabinoids administration have been described, the precise neurobiological mechanisms underlying cannabinoid addiction in vulnerable individuals remain largely unknown. At the present moment, animal models of cannabinoid addiction have not been yet developed, and there is an urgent need of an accurate animal model to disentangle the neurobiological correlates of addiction to cannabinoids.

Animal models of drug exposure allow to investigate brain long-lasting changes produced by drugs of abuse. Non-contingent drug administration animal models were firstly developed to evaluate short and long-lasting changes after exposure to a drug (Panlilio et al., 1998). In contrast, contingent operant self-administration models allow to directly evaluate a drug’s reinforcing property with a high predictive value to model voluntary drug consumption in humans. The current model provides for the first time an animal model of cannabinoid addiction that recapitulates the diagnostic criteria used in DSM-5 to define this human disorder (Deroche-Gamonet et al., 2004; Belin and Everitt, 2008; Maldonado et al., 2021). Indeed, our animal model has been generated based on the three main behavioral hallmarks of addiction that englobe DSM-5 addiction criteria (Supplementary Figure S1): 1) persistence of response (criteria 6 and 7 of DSM-5), 2) motivation for the drug (criteria 9 and 10 of DSM-5), 3) compulsive-like behavior defined as a disruption of inhibitory control despite negative consequences (criterion 11 of DSM-5). Our model also measures extinction reactiveness and reinstatement, parameters closely related with craving, another addiction diagnostic criteria of the DSM-5, as well as other phenotypic traits of predictive value for the development of cannabinoid addiction.

In this study, we used the synthetic cannabinoid agonist WIN 55,212–2, which is a potent full agonist of cannabinoid receptor 1 (CB1R) (Compton et al., 1992), to generate a model of cannabinoid addiction (Maldonado et al., 2011). Previous studies tried to obtain an operant intravenous (iv) self-administration of delta9-tetrahydrocannabinol (THC), which is the primary psychoactive component of the *cannabis sativa* plant. However, few studies were able to maintain a model with persistent, dose-related behavior regarding THC iv self-administration (Justinova et al., 2003; Spencer et al., 2018), as well as vapor self-administration (Freels et al., 2020), showing the necessity of alternative methods. In contrast to THC, mice achieved a reliable operant iv self-administration with the synthetic cannabinoid agonist WIN 55,212–2 (Martellotta et al., 1998; Mendizábal et al., 2006), although this cannabinoid has not yet been used to validate a model of cannabinoid addiction. This study aimed to validate for the first time a reliable mouse model of cannabinoid addiction by using WIN 55,212–2 iv operant self-administration.

This behavioral protocol can be combined with multiple neurochemical, electrophysiological, optogenetic, and chemogenetic manipulations to decipher the neurobiological mechanisms involved in cannabinoid addiction. For this purpose, we chemogenetically silenced the prelimbic (PL) -nucleus accumbens (NAc) pathway in our mouse model through a Designer Receptors Exclusively Activated by Designer Drugs (DREADD) approach, a pathway closely involved in the development of addictive behavior (Domingo-Rodríguez et al., 2004; Compton et al., 2022).

## 2 Materials and equipment

### 2.1 Animals

Eight weeks old male C57BL/6J mice (n = 30) (Charles River, France) were housed individually with food and water available *ad libitum* in controlled laboratory conditions (21ºC ± 1°C, 55% ± 10%). Mice were tested during the first hours of the dark phase of a reversed light/dark cycle (lights off at 8:00 a.m. and on at 20:00 p.m.). Body weight and food intake were monitored throughout the entire experiment. All animal procedures were approved by the local ethical committee (Comitè Ètic d'Experimentació Animal-Parc de Recerca Biomèdica de Barcelona, CEEA-PRBB, agreement N°9687) and conducted in strict conformity with the guidelines of the European Communities Council Directive (2010/63/EU) regulating animal experimentation, in the animal facility at Universitat Pompeu Fabra-Barcelona Biomedical Research Park (UPF-PRBB; Barcelona, Spain). All the experiments were performed under blind and randomized conditions. The male sex was chosen accordingly with the previous literature that has validated the operant WIN 55,212–2 self-administration model only in males (Martellotta et al., 1998; Mendizábal et al., 2006; Mancino et al., 2015; Domingo-Rodriguez et al., 2020; Martín-García et al., 2020; García-Blanco et al., 2022).

### 2.2 Drugs

WIN 55,212–2 [(R)-(+)-WIN 55,212–2 mesylate salt, Sigma-Aldrich, U.S.A.] was dissolved in one drop of Tween 80 (TWEEN 80, Sigma-Aldrich, U.S.A.) and then diluted in heparinized (1%) sterile saline solution and made available at two different doses: 0.1 mg/kg for intraperitoneal (ip) injection 24 h before the first operant session and 12.5 μg/kg/infusion for the self-administered iv infusions. The preparation was covered from the light and stored at room temperature. After each self-administration session, 0.05 mL of sodic heparin (Hospira 5%, Hospira, Pfizer) was applied through the iv catheter to avoid coagulation and obstruction of the latter. Thiopental sodium (5 mg/mL, Braun Medical S.A.) was dissolved in distilled water and injected in a volume of 0.05 mL through the iv catheter to evaluate catheter patency.

### 2.3 Operant self-administration apparatus

Experiments were performed in mouse operant chambers (model ENV-307A-CT, Med Associates Inc., Georgia, VT, U.S.A.) equipped with two nose-pokes, one randomly selected as the active hole and the other as the inactive hole. A house light was located on the chamber's ceiling, and two stimuli lights (cues) were placed one inside the active hole and the other above it. Nose-poking on the active hole resulted in the delivery of one WIN 55,212–2 infusion (under the associated schedule) paired with the activation of the stimulus light located above the active hole, while nose-poking on the inactive hole had no consequences. The chambers were made of aluminum and acrylic and placed inside sound- and light-attenuated boxes equipped with fans providing ventilation and white noise. The chamber’s floor was a grid made with metal bars that could conduct electrical current when performing the shock test. WIN 55,212–2 (12.5 μL/kg/infusion) was delivered in a volume of 23.5 μL over 2 s *via* a syringe firmly attached to a micro infusion pump (PHM-100A, Med-Associates, Georgia, VT, U.S.A.) and connected with flexible polymer tubing (0.96 mm outer diameter, Portex Fine Bore Polythene Tubing, Portex Limited, Kent, England) to a single channel liquid swivel (375/25, Instech Laboratories, Plymouth Meeting, PA, U.S.A.) and the mouse iv catheter.

## 3 Methods

### 3.1 WIN 55,212–2 self-administration

#### 3.1.1 Jugular vein catheterization

Mice (n = 30) were anesthetized by ip injection (0.2 mL/10 g of body weight) of ketamine hydrochloride (75 mg/kg of body weight, Ketamidor, Richterpharma ag, Austria) and medetomidine hydrochloride (1 mg/kg of body weight, Domtor, Esteve, Spain) dissolved in 0.9% sterile physiological saline and then implanted with indwelling iv silastic catheters in the right jugular vein, as previously described (Martín-García et al., 2009). Briefly, a 6 cm long silicone tubing (0.3 mm inner diameter, 0.6 mm outer diameter; Silastic, Dow Corning, Houdeng-Goegnies, Belgium) was adapted to a 22-gauge steel cannula (Semat, Herts, England) curved at a right angle and embedded in a dental cement disk (Dentalon Plus, Heraeus Kulzer, Germany) with a nylon mesh underneath. The catheter tubing was inserted 1.1 cm into the right jugular vein and attached with a suture. The remaining tubing was inserted subcutaneously (sc) to the cannula, exiting at the midscapular region. All incisions were sutured and coated with a local analgesic (Blastoestimulina, Almirall, Spain). Post-surgery procedure consisting of an ip injection of antibiotic (1 mg/kg of body weight, Gentamicine, Genta-Gobens, Laboratorios Normon, Spain), a sc injection of analgesic (mixture of glucose serum (GlucosaVet, B. Braun Vet Care, Spain) and meloxicam (2 mg/kg of body weight, Metacam, Boehringer Ingelheim, Rhein) and a sc injection of an anesthesia reversor, atipamezole hydrochloride (2.5 mg/kg of body weight, Revertor, Virbac, Spain), was applied all dissolved in 0.9% sterile physiological saline. Mice were allowed to recover for 3 days, with follow-up analgesics, prior to the initiation of the self-administration sessions. The patency of iv catheters was assessed by a thiopental sodium test at the end of the self-administration experimental sequence. The mouse was removed from the experiment if prominent signs of anesthesia were not observed immediately after injection (n = 1 in this study).

#### 3.1.2 WIN 55,212–2 self-administration training

**FIGURE 1:**
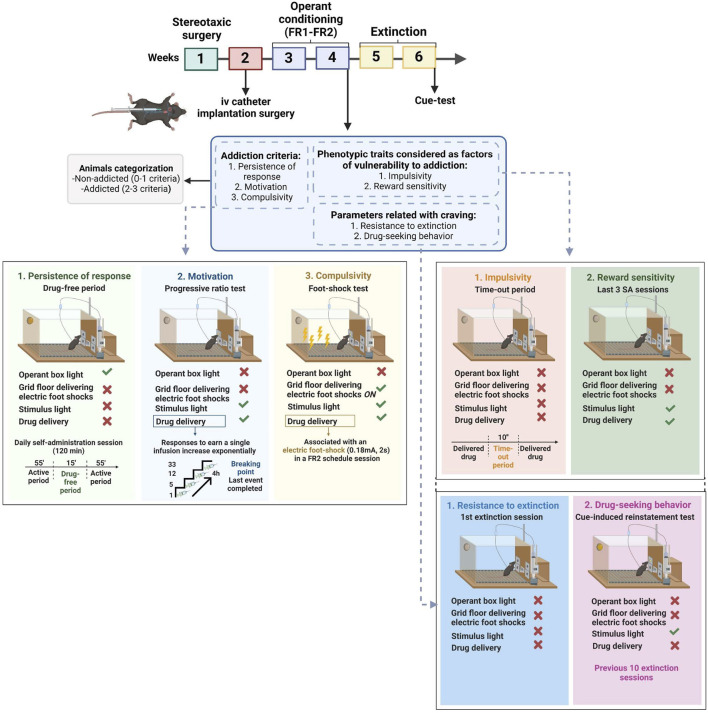
Complete experimental protocol of our mouse model of operant WIN 55,212–2 intravenous self-administration.

The operant model was applied accordingly to previous drug self-administration paradigms (Mendizábal et al., 2006; Vallée et al., 2014; Martín-García et al., 2016). To avoid the aversive effects of the drug's first administration, mice received an ip injection of WIN 55,212–2 (0.1 mg/kg) only 24 h before the first self-administration session (Valjent and Maldonado, 2000; Mendizábal et al., 2006; Vallée et al., 2014). Subsequently, mice (n = 29) were trained to acquire an operant self-administration conditioning maintained by iv infusions of WIN 55,212–2. The schedule was a fixed ratio (FR) 1 schedule of reinforcement during 5 consecutive sessions, followed by a progression to FR2 for another 5 sessions. All sessions were performed at the same time and scheduled every day. Each daily self-administration session was started with a priming injection of the drug (0.0125 mg/kg/infusion) automatically delivered iv through the catheter when the session was initiated (Mendizábal et al., 2006; Martín-García et al., 2016; Flores et al., 2020), followed by two 55 min active periods separated by a 15 min drug-free period for a total duration of 125 min. The initiation of each session was signaled by turning on the house light only during the first 3 s. The cue lights, together with the noise of the infusion pump, acted as environmental cues signaling the drug infusion. A 10 s time-out period was fixed after each drug delivery, during which the cue light was off, and no reward was provided after responding to the active nose-poke. Responses to the active and inactive holes and all responses executed during the time-out were recorded. During the drug-free period, no reinforcer nor cue was delivered, signaled by the activation of the house light. The session was concluded after 50 reinforcers were delivered or after 125 min, whichever occurred first. The acquisition of the self-administration behavior was achieved when the three following conditions were met: 1) mice maintained 80% of stability in three consecutive training sessions, meaning that the variance during these 3 days was 20% or less, 2) at least 75% responding on the active hole, and 3) a minimum of five reinforcers per session. After each session, mice were brought back to their home cages (Figure 1).

#### 3.1.3 Three addiction criteria

The development of addictive-like behaviors was evaluated at the end of the training sessions based on three addiction-like criteria that summarize the addiction hallmarks according to the DSM-5 (Deroche-Gamonet et al., 2004; Piazza and Deroche-Gamonet, 2013; Domingo-Rodríguez et al., 2022). The addiction score developed was then attributed based on the results of these three criteria, each determined by the respective behavioral test:

##### 3.1.3.1 Persistence to response

The number of non-reinforced active responses during the 15 min drug-free period was measured as persistence of drug-seeking behavior. Mice were scored on the three consecutive days before the progressive ratio (PR).

##### 3.1.3.2 Motivation

The PR schedule of reinforcement evaluated the motivation towards the reinforcer. The responses required to receive one drug infusion escalated following this series: 1, 5, 12, 21, 33, 51, 75, 90, 120, 155, 180, 225, 260, 300, 350, 410, 465, 540, 630, 730, 850, 1000, 1200, 1500, 1800, 2100, 2400, 2700, 3000, 3400, 3800, 4200, 4600, 5000, and 5500. The breaking point, the maximal number of responses mice perform to obtain one infusion defined as the motivation value, corresponds to the last ratio completed. The duration of the PR session was maximum 4 h or until mice stopped responding to any nose-poke within 1 h.

##### 3.1.3.3 Compulsivity

Resistance to punishment, now defined as compulsive-like behavior, corresponded to the maintenance of active responding behavior despite its association with a negative consequence. It was measured by the total number of shocks obtained in a 50 min shock test, during which each drug delivered was associated with a foot-shock-induced punishment. This shock session was performed after a stabilizing FR2 self-administration session following the PR test. Mice were placed in a different operant box than the one regularly used for the operant sessions. Then mice underwent an FR2 self-administration schedule of reinforcement for 50 min with two scheduled changes: after one active response, mice received an electric foot-shock (0.18 mA, 2 s), while after the second response, the electric foot-shock was paired with the drug delivery and the associated cue light. In parallel, if the second response was not completed within a min after completing the first response, the sequence was reinitiated.

#### 3.1.4 Establishment of mice subpopulations

After the three behavioral tests were performed, mice were categorized into addicted and non-addicted animals based on the number of positive criteria achieved. A mouse was considered to be positive for an addiction-like criterion when the score of the behavioral test was equal to or beyond the 75th percentile of the normal distribution of the saline group. Mice that achieved 2 or 3 criteria were considered addicted and categorized as vulnerable, whereas those reaching 0 or 1 criterion were considered non-addicted and categorized as resilient.

#### 3.1.5 Extinction and parameters related with craving

Only mice with patent catheters that reached all acquisition criteria continued to the extinction phase. After thiopental testing, mice were allowed to rest for 1 day, during which they underwent a 2-h locomotion test in individual locomotor activity boxes (10.8 × 20.3 × 18.6 cm, Imetronic, Pessac, France) equipped with infrared sensors to detect locomotor activity and an infrared plane to detect rearings.

During the extinction period, neither WIN 55,212–2 infusions, priming infusions, nor the associated environmental cues were delivered after nose-poking on the active hole. Mice were exposed to 2-h daily sessions for 10 consecutive days in the same operant chamber as the self-administration sessions. During this period, mice reached the extinction criterion when responses to the active nose-poke were < 35% of the mean responses obtained during the last 3 days of WIN 55,212–2 self-administration across three consecutive extinction sessions. Only mice that achieved the extinction criterion were evaluated for the following. Two parameters related with craving were evaluated before and after this extinction period:

##### 3.1.5.1 Resistance to extinction

Number of active responses in 2 h during the first extinction session. Animals with significant sensitivity to drug withdrawal will increase their resistance to extinction by increasing the number of active nose-pokes to seek the drug when access is prevented.

##### 3.1.5.2 Drug-seeking behavior

The day after achieving the extinction criterion, we performed a single cue-induced reinstatement session in the same operant chamber, in order to test reinstatement of drug-seeking behavior upon exposure to the environmental stimuli after a period of abstinence. The cue test was conducted under the same conditions used in the acquisition phase, except that active responding was not reinforced by the drug. This meant that mice were subject to a 90-min FR2 session, where the first 60 min were similar to an extinction session but in the last 30 min, nose-poking on the active hole resulted in the presentation of all the environmental cues associated (cue light, pump noise, and priming injection light) but not the delivery of WIN 55,212–2 (Martín-García et al., 2016; García-Blanco et al., 2022).

#### 3.1.6 Behavioral tests to evaluate addiction-like phenotypic traits

Two additional phenotypic traits were also evaluated as factors of vulnerability to addiction:

##### 3.1.6.1 Impulsivity

The number of non-reinforced active responses during the time-out periods (10 s) after each WIN 55,212-2 delivery was measured as impulsivity-like behavior, which indicated the inability to stop a response once it is initiated. The three consecutive days before the PR test were considered for this criterion.

##### 3.1.6.2 Sensitivity to reward

The number of reinforcers obtained in 2-h sessions during the last three consecutive FR2 operant conditioning sessions maintained by WIN 55,212–2. Animals with higher levels of sensitivity to reward will obtain a higher number of reinforcers.

### 3.2 DREADD approach: Surgery and viral vector microinjection

The adeno-associated viral (AAV) vectors used were: AAV-hM4Di-DREADD (AAV8-hSyn-DIO-hM4D(Gi)-mCherry, 1.21E+13 gc/mL) and AAV-retrograde-Cre-EBFP (AAV pmSyn1-EBFP-Cre; 8.2E+12 gc/mL) (Viral Vector Production Unit, Universitat Autònoma de Barcelona).

Mice were anesthetized by ip injection (0.2 mL/10 g of body weight) of ketamine hydrochloride (75 mg/kg of body weight, Ketamidor, Richterpharma ag, Austria) and medetomidine hydrochloride (1 mg/kg of body weight, Domtor, Esteve, Spain) dissolved in 0.9% sterile physiological saline and located into a stereotaxic apparatus to receive the intracranial AAV injections. All injections were performed through a bilateral injection cannula (33-gauge internal cannula, Plastics One, United Kingdom) connected *via* a polyethylene tubing (PE-20, Plastics One, United Kingdom) to a 10 μL microsyringe (Model 1701 N SYR, Cemented NDL, 26 s GA, 2 in point style 3, Hamilton company, NV). The displacement of an air bubble along the tubing connecting the syringe to the injection needle was utilized to monitor the microinjections. For the precise inhibition of the PL-NAc pathway, two bilateral injections were performed, one targeting the PL and the other the NAc core. Mice were injected with 0.2 μL per site of the AAV-hM4Di-DREADD into PL (rate infusion of 0.05 μL/min) and 0.4 μL per site of the AAV-retrograde-Cre-EBFP into the NAc core (rate infusion of 0.10 μL/min). After infusion, the injection cannula was left untouched for an additional 10 min to permit the fluid to diffuse and prevent reflux, and then slowly withdrawn. A heating pad was used to preserve the body temperature at 35°C. The coordinates used followed the Paxinos and Franklin atlas (Franklin and Paxinos, 1997): (PL) AP +2.10 mm, L ±0.3 mm, DV -2.3 mm; (NAc core) AP +1.94 mm, L ±1 mm, DV -4.6 mm.

Clozapine-N-oxide (CNO*,* Enzo Life Sciences, NY), a behaviorally inert drug, was administered *via* Alzet osmotic minipumps (Model 2004; Alzet, Cupertino, CA) previously filled with either CNO (diluted in 0.9% sterile saline; 5 mg/mL) or physiological saline solution. Minipumps were sc implanted in the lower back of each mouse during the jugular vein catheterization surgery. The osmotic minipumps delivered CNO by osmosis at a constant sc flow rate of 0.25 μL/h for 15 days (Domingo-Rodríguez et al., 2020; Martín-García et al., 2020).

### 3.3 Statistical analysis

#### 3.3.1 Statistical analysis of behavioral data

The number of animals (n) in each experimental condition is indicated in the figure legends. All statistical comparisons were performed with SPSS (IBM, version 25). Comparisons between two groups were performed by Student’s t-test or U Mann-Whitney test according to the distribution defined by the Kolmogorov-Smirnov normality test. ANOVA with repeated measures was used when necessary to test the evolution over time, followed by *post hoc* analysis (Fisher LSD) for multiple group comparison. The Pearson correlation coefficient was performed to analyze the relationships between values in each addiction-like criterion and the final criteria achieved. The chi-square analysis were used to compare the percentage of addicted and non-addicted mice. Results were expressed as individual values with the median and the interquartile range or with the mean ± S.E.M, which is specified in the figure legend. A *p*-value <0.05 was applied to determine statistical significance.

The sample size was calculated based on the power analysis. The significance criterion (alpha) was set at 0.050, and the statistical test utilized was a two-sample *t*-test. With the sample size of 13–16 mice per group, our studies achieved a power superior to 80%. Supplementary tables (Supplementary Tables S1–S2) provide a complete report of the statistical results for the data described in the figures.

#### 3.3.2 Principal component analysis

The principal component analysis (PCA) was performed to evaluate the multidimensional behavioral data by reducing it to fewer dimensions in order to observe trends, clusters, and outliers. PCA and varimax rotation were conducted using the three addiction-like criteria, the two parameters related to craving and the two phenotypic traits considered as vulnerability factors of addiction to dimensionality reduce them to the minimum number of components that best explain and maximize the variance present in the data set. An eigenvalue greater than 1 was set as selecting components criterion (Field, 2018).

## 4 Results

### 4.1 WIN 55,212–2 self-administration led to the development of an addictive-like phenotype in mice

**FIGURE 2:**
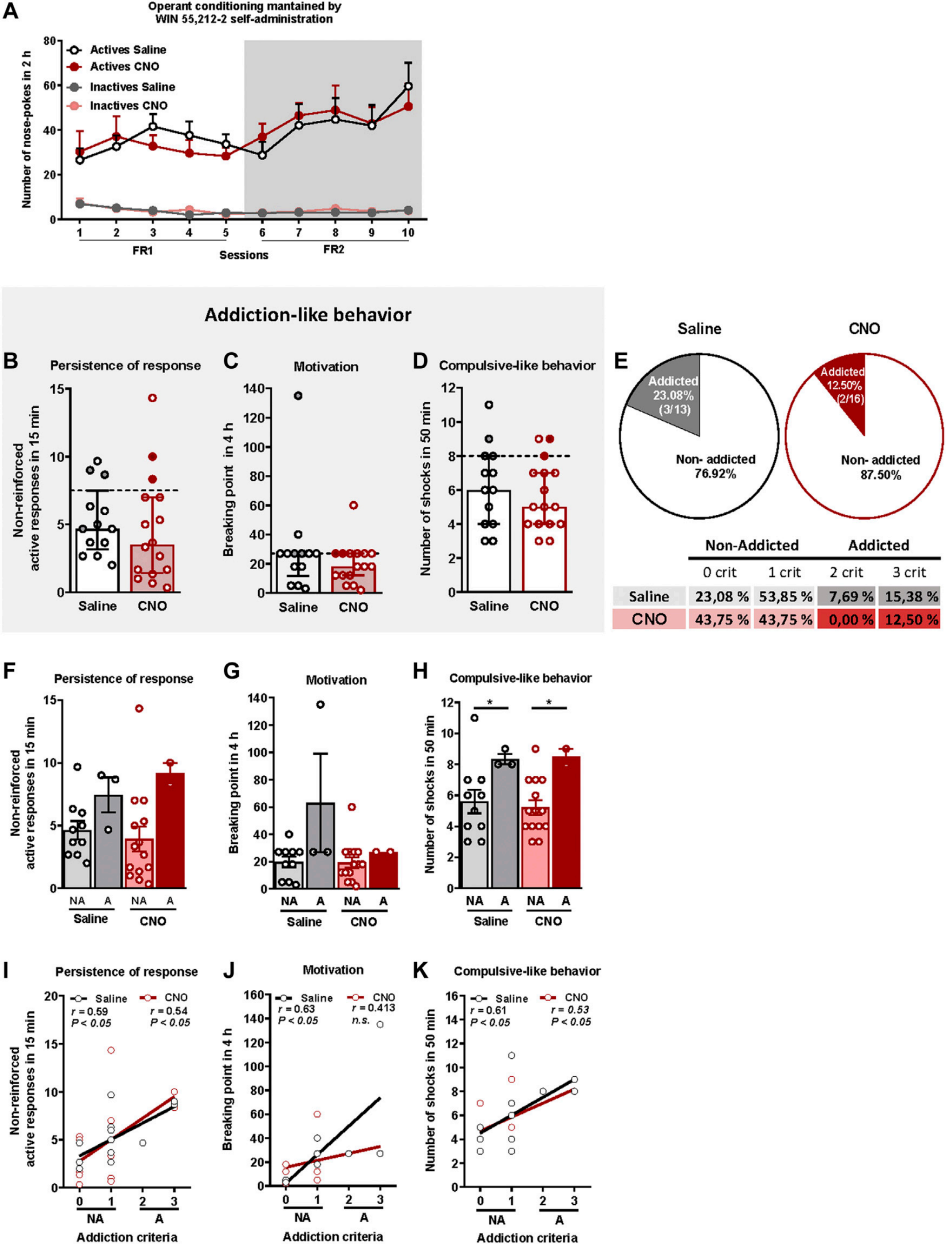
WIN 55,212–2 operant self-administration led to the development of an addictive-like phenotype in mice. **(A)** Similar number of total active and inactive nose-pokes performed by CNO- and saline-treated groups during 2 h of operant self-administration maintained by intravenous infusions of WIN 55,212–2 in both FR1 and FR2 schedules of reinforcement (mean ± S.E.M., repeated measures ANOVA). **(B–D)** Mice present similar responses in the three addiction-like criteria tests (individual data with median and interquartile range): **(B)** Persistence to response: Number of responses to the active nose-poke during the 15 min drug-free period (Student’s t-test). **(C)** Motivation: Breaking point determined during a 4 h progressive schedule of reinforcement represents the maximal number of responses that an animal is able to do to obtain one drug infusion (U Mann-Whitney). **(D)** Compulsivity: Number of shocks received following the schedule described in the *Materials and Methods* section, reflecting the compulsivity level of each group (Student’s t-test). The dashed horizontal line indicates the 75th percentile of the distribution of the group, used as the threshold to consider a mouse positive for one criterion. Addicted mice are represented in grey- and red-filled circles. **(E)** Percentage of mice categorized as addicted (Chi square). **(F–H)** Behavioral tests of the three addiction-like criteria showing increased compulsivity in addicted mice compared to non-addicted but similar persistence to response and motivation (individual data with mean ± S.E.M., U Mann-Whitney, *p < 0.05). **(I–K)** Pearson correlations between individual values of addiction-like criteria and **(I)** non-reinforced active responses in 15 min, **(J)** breaking point in 4 h and **(K)** number of shocks in 50 min (Saline-treated mice: n = 13; CNO-treated mice: n = 16; statistical details are included in Supplementary Table S1).

We have developed for the first time a mouse model of cannabinoid addiction by using WIN 55,212–2 self-administration. In addition, we have evaluated in this model the possible involvement of the PL-NAc pathway in the development of this addictive behavior (Figure 1). Saline and CNO-treated mice were trained to acquire an operant self-administration sustained by iv infusions of WIN 55,212–2 (Figure 2A). The percentage of animals that achieved acquisition criteria of stability, discrimination, and number of reinforcers was 44.83% in the saline group and 55.17% in the CNO group (chi-square, C-S = 0.69, *n.s.*), with a progressive increase in the number of active nose-pokes across sessions (Repeated Measures ANOVA, [*F*_(1,54)_ = 27.76 in FR1, *F*_(1,54)_ = 0.01 in FR2, *p < 0.001,* DMS Actives vs. Inactives: *p < .001]*). No significant differences were found in active and inactive nose-pokes between CNO- and saline-treated mice in both FR1 and FR2 schedules, suggesting similar levels of operant conditioning maintained by WIN 55,212–2 (DMS Actives/Inactives CNO vs. Actives/Inactives Saline: *n.s.*).

The addiction score was calculated after the operant training using the three addiction-like criteria, as explained above. Extreme subpopulations of mice that present a high persistence of response, motivation and compulsivity, were revealed in both saline and CNO groups. Specifically, 26.1% (23.08% saline, 18.75% CNO), 51.7% (61.54% saline, 43.75% CNO) and 24.1% (30.77% saline, 18.75% CNO) of mice surpassed each criterion's threshold, suggesting the potential development of addictive-like behaviors after the chronic operant training. No significant differences were found between CNO- and saline-treated mice in persistence of response, motivation or compulsive-like behavior (Figures 2B–D). In the saline group, 23.08% (3/13) were considered addicted whereas 12.50% (2/16) were considered addicted in the CNO group (chi-square = 3.77, *n.s*., Figure 2E). Addicted mice showed a strong tendency for higher persistence of response compared to non-addicted in both saline and CNO groups and only for the saline-treated animals in the motivation (Figures 2F,G), whereas a significantly higher compulsive-like behavior was observed for addicted mice compared to non-addicted mice regardless of the treatment (U Mann-Whitney test, U = 3,000 for NA vs. A saline and U = 1,500 for NA vs. A CNO, *p < 0.05*, Figure 2H), Moreover, positive correlations were found between the number of criteria achieved and the severity of each criterion in both CNO- and saline-treated mice for all addictive-like criteria except for motivation in CNO-treated mice (Pearson correlations, *p < 0.05*, Figures 2I–K).

**FIGURE 3:**
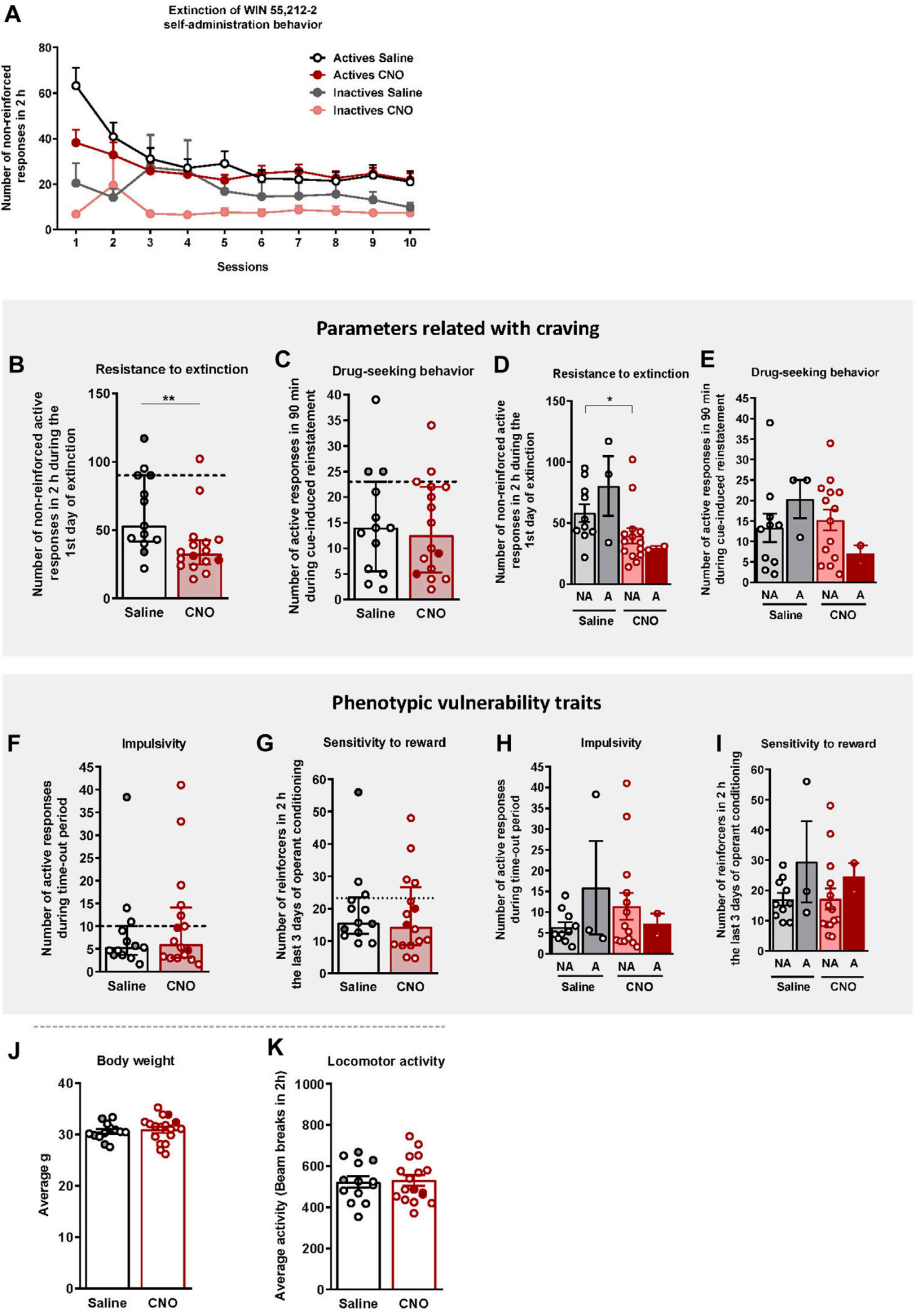
WIN 55,212–2 operant self-administration effects on two parameters related with craving and two phenotypic vulnerability traits to addiction-like behavior in mice. **(A)** Extinction pattern of the WIN 55,212–2 operant self-administration behavior (mean ± S.E.M., repeated measures ANOVA). **(B–C)** Behavioral tests of the two parameters related with craving (individual data with median and interquartile range): **(B)** Resistance to extinction: Number of responses to the active nose-poke during the first 2-h extinction session is significantly higher in saline-compared to CNO-treated mice (U Mann-Whitney, **p < 0.01). **(C)** Drug-seeking behavior measured by the cue-induced reinstatement after abstinence: Number of active responses performed during the 90 min cue-induced drug-seeking test performed after extinction (Student’s t-test). The dashed horizontal line indicates the 75th percentile of distribution of the group, used as the threshold to consider a mouse positive for one criterion. Addicted mice are represented in grey- and red-filled circles. **(D–E)** Behavioral tests of the parameters related with craving showing similar responses in cue-induced reinstatement between addicted and non-addicted mice (U Mann-Whitney), whereas a difference is observed between non-addicted mice in the 1st day of extinction (U Mann-Whitney, *p < 0.05) (individual data with mean ± S.E.M.). **(F–G)** Behavioral tests used to evaluate the two phenotypic traits considered to be factors of vulnerability to addiction-like behavior (individual data with median and interquartile range): **(F)** Impulsivity: Number of responses to the active nose-poke during the 10 s time-out period (U Mann-Whitney) **(G)** Sensitivity to reward: Number of reinforcers performed to the active nose-poke during the 2 h of the last three sessions of self-administration (Student’s t-test). The dashed horizontal line indicates the 75th percentile of distribution of the group, used as the threshold to consider a mouse positive for one criterion. Addicted mice are represented in grey- and red-filled circles. **(H–I)** Behavioral tests of the phenotypic traits showing similar responses in impulsivity and behavior during the last 3 days of operant training between addicted and non-addicted mice (U Mann-Whitney) (individual data with mean ± S.E.M.). **(J)** Body weight: Body weight was measured every week during the self-administration protocol (mean ± S.E.M.; Student’s t-test). **(K)** Locomotor activity: Activity was measured by the number of beam breaks during a 2 h test (mean ± S.E.M.; Student’s t-test). (Saline-treated mice: n = 13; CNO-treated mice: n = 16; statistical details are included in Supplementary Table S2).

After FR1 and FR2 training, mice underwent 10 sessions of extinction (Figure 3A). Both groups extinguished the self-administration behavior similarly (Repeated Measures ANOVA, [Active lever presses: *F*_(1,27)_ = 0.15, *n.s.*, Inactive lever presses: *F*_(1,27)_ = 0.38, *n.s.]*), despite a higher number of reinforcers obtained during the first session in saline-treated mice compared to the CNO group (U Mann-Whitney test, U = 42,000, *p < 0.01*, Figure 3B). Responses to the active nose-poke declined over time until reaching 58% and 43.7% decrease of the active nose-poke in the last session compared to the last operant session for the saline and CNO groups, respectively.

Animals that responded < 35% of the mean responses performed during the last 3 days of WIN 55,212–2 self-administration across three consecutive extinction sessions acquired the extinction criteria (30.77% of saline and 12.50% of CNO mice, chi-square = 1.92, *n.s*.). Resistance to extinction, measured on the first day of extinction, was significantly lower in the CNO group compared to saline-treated mice (U Mann-Whitney test, U = 42.000, *p < 0.01*, Figure 3B). In contrast, no significant differences were obtained between groups for the cue-induced reinstatement of drug-seeking behavior (Figure 3C). Non-addicted mice in the saline group showed higher levels of response in the first extinction session compared to non-addicted mice in the CNO group (U Mann-Whitney test, U = 32.000, *p < 0.*05, Figure 3D), while no significant differences were obtained for the drug-seeking behavior (Figure 3E).

### 4.2 WIN 55,212–2 self-administration effects on phenotypic vulnerability traits to addiction-like behavior in mice

Two phenotypic traits considered as vulnerability factors to addiction-like behavior, impulsivity and sensitivity to reward, were also evaluated. No significant differences were found between CNO (mean ± S.E.M.: 10.85 ± 2.85; 18.08 ± 3.11) and saline-treated mice (mean ± S.E.M.: 8.56 ± 2.66; 19.90 ± 3.42), neither in impulsivity nor reward sensitivity respectively (Figures 3F,G). No significant differences between groups were neither observed when the population of saline and CNO groups was divided into addicted and non-addicted (Figures 3H,I).

To confirm that CNO treatment did not produce any effect that could bias our self-administration results (Roth, 2016), the body weight, locomotor activity, and food intake of mice were monitored throughout the experiment. No significant differences were observed between saline- and CNO-treated mice in terms of body weight (Figure 3J), and food intake (*data not shown*) across the entire experiment. Moreover, no significant differences were observed between groups in the locomotor activity (Figure 3K), sustaining the absence of side effects of CNO treatment.

### 4.3 Principal component analysis of cannabinoid addiction through WIN 55,212–2 self-administration

**FIGURE 4:**
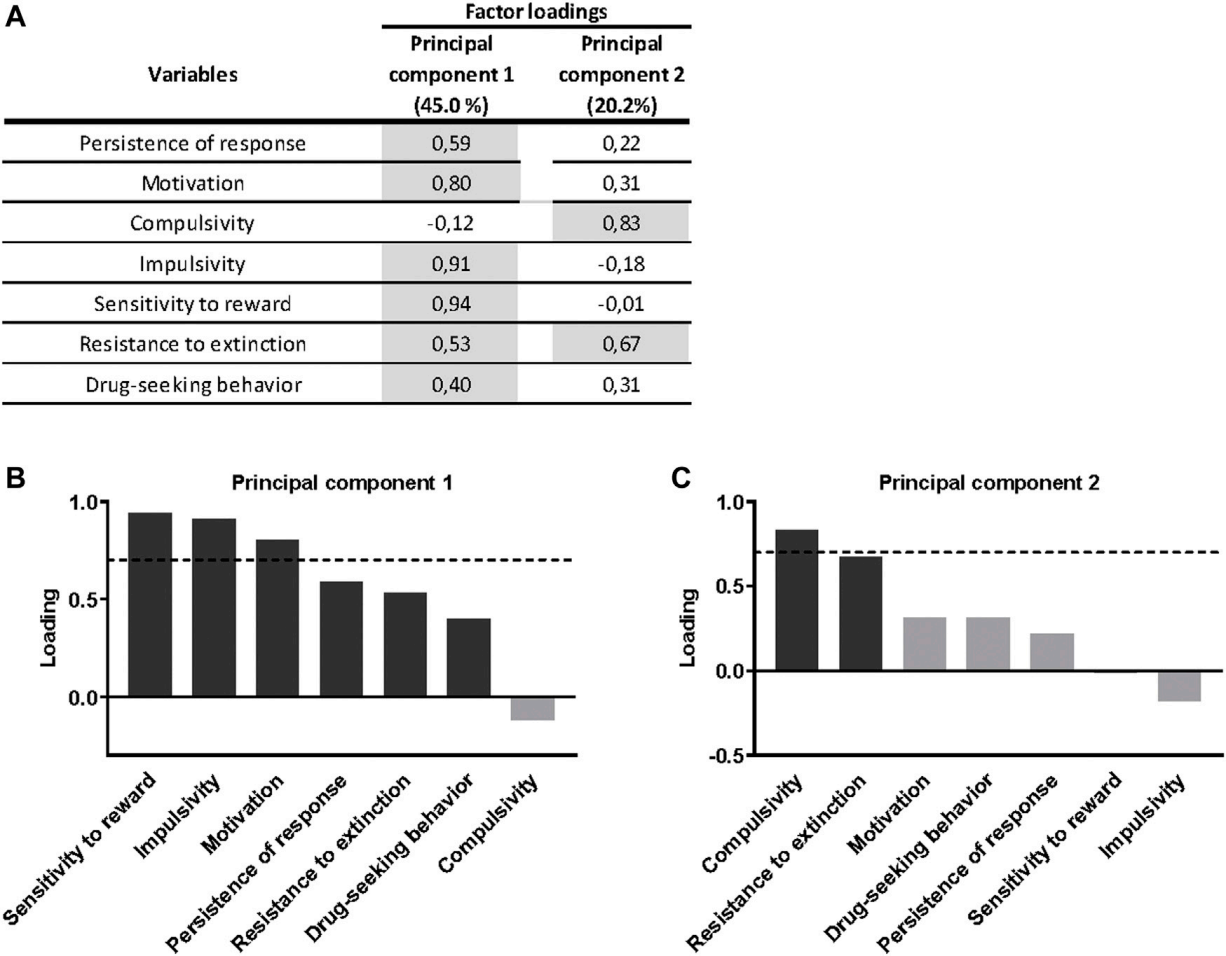
Principal component analysis of cannabinoid addiction through WIN 55,212–2 operant self-administration. **(A)** Factor loadings of the principal component 1 (PC1) and principal component 2 (PC2) in all variables studied. **(B–C)** Order of factor loading of the different variables in PC1 and PC2. The dashed horizontal line marks loading greater than 0.7, mainly contributing to the component. In regards to the addiction criteria, a dissociation between motivation and persistence of response, mainly contributing to PC1, and compulsivity, mainly contributing to PC2, can be observed. Regarding parameters related to craving, drug-seeking behavior weighted more in the PC1, while resistance to extinction weighted more in the PC2, even though its influence is also present in the PC1. For the phenotypic traits, both weighted more in the PC1.

A principal component analysis was used to determine whether the behavioral outcomes previously described could be reduced to fewer dimensions that might display individual differences in cannabinoid addiction. All addiction criteria, parameters related with craving and phenotypic traits were taken into account. Principal component 1, which accounts for 45.0% of the variance (Figure 4A), has strong loadings (>0.7) from all behavioral variables except compulsivity. These traits are associated to the development of cannabinoid addiction and, therefore, they contribute to this development. The second principal component, which is orthogonal to component 1 and accounts for 20.2% of the variance, is comprised of two variables, the criteria of compulsivity and resistance to extinction. Interestingly, impulsivity participates more in the first component, while compulsivity is more critical in the second component (Figures 4B,C), resembling the sequential feature of the transition from impulsivity to compulsivity described in addiction. Finally, most of the phenotypic traits of vulnerability are in the same component suggesting similar neurological correlates.

### 4.4 Correlation heatmap of the variables of cannabinoid addiction criteria, parameters related with craving and vulnerability phenotypic traits

**FIGURE 5:**
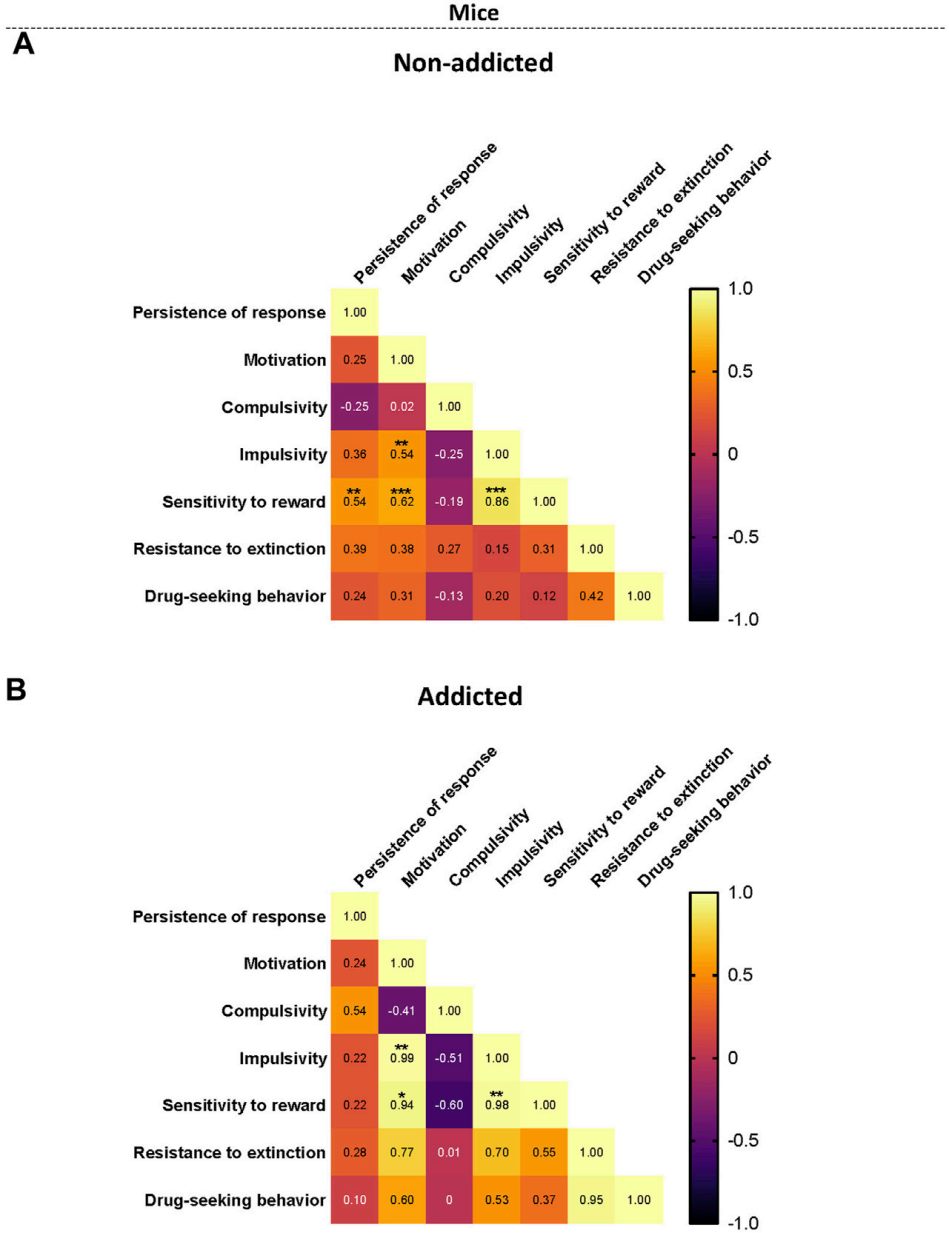
Correlation heatmaps of the variables of cannabinoid addiction criteria, parameters related to craving, and phenotypic vulnerability traits. **(A–B)** Pearson correlations between the three addiction-like criteria, the two parameters related with craving and the two phenotypic traits in both **(A)** non-addicted and **(B)** addicted groups. (*p < 0.05, **p < 0.01, ***p < 0.001).

When representing the addiction-like criteria, the parameters related with craving and the phenotypic traits into a heat map, non-addicted animals revealed significant correlations between persistence to response and sensitivity to reward (r = 0.54, *p < 0.01*), motivation and sensitivity to reward (r = 0.62, *p < 0.001*), impulsivity and sensitivity to reward (r = 0.86, *p < 0.001*), and motivation and impulsivity were significant (r = 0.54, *p < 0.01*) (Figure 5A). In addicted animals, the significant correlations between motivation and impulsivity (r = 0.99, *p < 0.01*), motivation and sensitivity to reward (r = 0.95, *p < 0.05*), and impulsivity and sensitivity to reward (r = 0.98, *p < 0.01*) were maintained (Figure 5B). Theses results were in agreement with the results obtained in the PCA. Interestingly, compulsivity and impulsivity showed a negative correlation (non-significant) in coherence with the differential load of each variable in the PCA.

## 5 Discussion

Cannabis addiction, defined in the 5^th^ edition of the DSM as cannabis use disorder, is a major concern worldwide, and its neurobiological substrate is still largely unknown. The absence of animal models that recapitulate the hallmarks of cannabinoid addiction has impaired the animal research focused on this disorder. In this study, we developed for the first time an animal model of cannabinoid addiction that recapitulates the main diagnosis criteria of this disorder defined on the DSM-5 based on an operant paradigm of WIN 55,212–2 iv self-administration.

Drug self-administration paradigms based on their positive reinforcement effects can be used to model certain aspects of the human addictive behavior (Markou et al., 1999). However, previous studies have demonstrated the complexity in modeling cannabis self-administration compared to other drugs due to the difficulty to obtain a reliable self-administration of cannabinoids in animals (Panlilio and Justinova, 2018). Operant self-administration of THC is difficult to be reliably maintained in animals (Tanda and Goldberg, 2003). Only a few number of studies in squirrel monkeys (Justinova et al., 2003) and rats (Spencer et al., 2018; Freels et al., 2020) have been able to show that THC can maintain self-administration (intravenous or vapor) without a previous history of exposure to other drugs. The failure of THC to act as reinforcer in animal studies has been related to its delayed onset of pleasurable pharmacological effects, the long duration of its pharmacological and behavioral effects, and its partial agonist profile (Tanda and Goldberg, 2003). Due to these difficulties, most of the operant self-administration studies have used synthetic cannabinoids. WIN 55,212–2 is a potent synthetic cannabinoid with a shorter half-life than THC and a full agonist of the CB1R (Pertwee et al., 2010). These characteristics may explain the difference in reinforcing properties and the improving characteristics for operant self-administration models in comparison to THC (Maldonado et al., 2011). Several studies have achieved reliable operant paradigms to self-administer WIN 55,212–2 in mice and rats (Martellotta et al., 1998; Fattore et al., 2001; Mendizábal et al., 2006; Lefever et al., 2014). However, none of these self-administration studies has been used to generate a model of cannabinoid addiction.

In our study, we have established a model in which vulnerable mice present persistence to self-administer WIN 55,212–2, extremely high motivation to obtain WIN 55,212–2 infusions, and compulsivity to WIN 55,212-2-seeking and self-administer despite adverse consequences, which confirms the development of a cannabinoid addiction-like model based on iv infusions of WIN 55,212–2. These criteria allowed to separate two populations of mice with vulnerable and resilient phenotypes to develop cannabinoid addiction. Therefore, the establishment of this cannabinoid addiction mouse model using WIN 55,512–2 iv self-administration represents a pivotal tool for future research allowing to elucidate the neurobiological correlates underlying resilience and vulnerability to develop this disorder. These addiction-like hallmarks used to establish this cannabinoid addiction model have been extracted from an established rat model of cocaine addiction (Deroche-Gamonet et al., 2004), and have been repeatedly used as a reference to establish mice models of drug and food addiction (Martín-García et al., 2016; Domingo-Rodríguez et al., 2020).

A main problem in the treatment of addiction is the high rates of relapse to drug use after periods of abstinence (Venniro et al., 2016; Fredriksson et al., 2021). An important advantage of our cannabinoid addiction model is the possibility to evaluate two parameters closely related with craving and relapse, resistance to extinction and cue-induced reinstatement. Resistance to extinction measures an ‘extinction burst’ behavior typically seen in rodents during the first day of extinction (Cooper et al., 1987) that has been revealed with different drugs of abuse (Peltier et al., 2001; Shalev et al., 2002; Soria et al., 2008). The resistance to extinguish the operant behavior revealed in the present study suggests that mice had developed a reliable and persistent operant WIN 55,212–2 self-administration behavior, and this behavior has been reported to reflect a ‘craving-like’ state at the beginning of the extinction training (Fredriksson et al., 2021). Craving during abstinence has been suggested to be directly involved with the vulnerability to relapse (Venniro et al., 2016; Fredriksson et al., 2021). Reinstatement of drug seeking is typically assessed by the extinction-reinstatement model (Shaham et al., 2003; Weiss, 2010) and non-reinforced responding to the previously learned active nose-poke is the measure of drug seeking (Stewart and de Wit, 1987). In our model, we performed a cue-induced reinstatement procedure after extinction, in which re-exposure to conditioned cues when responding to the active nose-poke, cues that had been contingently paired with drug delivery during acquisition, reinstated drug seeking (Davis and Smith, 1976). Exposure to drug-associated cues can elicit drug desire and drug seeking, effects implicated both in the maintenance of ongoing drug use and inducing drug seeking after abstinence, which shows important resistance to extinction (Weiss and Volkow, 2022). However, some studies argue that human abstinence is often either forced or voluntary (self-imposed) (Venniro et al., 2016; Fredriksson et al., 2021). In fact, drug relapse and craving are commonly triggered not only by drug-associated cues, but also by acute exposure to the self-administered drug, stress and short-term or protracted withdrawal symptoms (Venniro et al., 2016; Fredriksson et al., 2021). Alternative animal models have been developed in which abstinence is not obtained by extinction training but through forced abstinence, mainly assessed by the incubation of drug craving model (Grimm et al., 2001) or by voluntary abstinence, achieved either by the introduction of negative consequences to ongoing drug-administration (Panlilio et al., 2003; Cooper et al., 2007), or of alternative non-drug reinforcers (Ahmed et al., 2013).

This model also evaluates two phenotypic traits related to addiction vulnerability factors. Impulsivity is a complex construct composed of motor impulsivity and choice impulsivity (Belcher et al., 2014). In our model, we have considered the non-reinforced active nose-pokes during the time-out periods to evaluate the motor impulsivity defined as motor disinhibition, as previously described (Domingo-Rodriguez et al., 2022), The impulsivity trait has been associated with drug addiction since it predicts the transition to compulsive drug intake (Belin and Deroche-Gamonet, 2012; Weafer et al., 2014), and neurobiological correlates underlying this phenotypic trait could reveal potential biomarkers and/or therapeutic targets for cannabinoid addiction. Reward sensitivity is associated with an increased probability of responding with a positive hedonic component involving pathways that have a crucial role in the rewarding properties of drugs of abuse (Koob and Volkow, 2016).

We have used this cannabinoid addiction model to evaluate the possible involvement of the PL- NAc glutamatergic pathway, which plays a crucial role in food addiction (Domingo-Rodríguez et al., 2020). This pathway is modulated by the endocannabinoid system and deletion of the CB_1_R of these glutamatergic neurons leads to a resilient phenotype to develop food addiction (Domingo-Rodríguez et al., 2020). CB1Rs are the main cannabinoid receptors involved in the development of cannabinoid addiction (Maldonado et al., 2011) and the inhibition of the activity of the PL-NAc pathway may modify the development of cannabinoid addiction. However, CNO activation of the inhibitory DREADDs expressed in the PL-NAc pathway did not alter the addictive-like behavior regarding the addictive criteria and phenotypic vulnerability traits. However, the resistance to extinction was decreased in CNO-treated animals, suggesting a protective effect of this manipulation on the craving-like state. It is important to underly that the patency of the catheters limited the protocol to 2 weeks. Thus, the time between when the minipumps filled with CNO were implanted and the performance of the addictive-like behavioral tests was merely 15 days. Hence, the CNO had only this short period of time to be released from the minipump and act on the DREADD to inhibit the pathway. We hypothesize this to be the reason for the absent effect of this pathway’s inhibition, as the long-term action of the CNO and the hM4Di DREADDs in inhibiting a neuronal pathway has been confirmed in many studies (Domingo-Rodríguez et al., 2020; Martín-García et al., 2020).

The influence of the environment is key in the onset of consumption as well as the maintenance of cannabinoid addiction. Indeed, environments with high levels of social stressors, lack of opportunities, easy accessibility to drugs, and lack of alternative reinforcers, lead to an elevated risk for addiction development (Volkow et al., 2019). Consequently, the biggest shortcoming of this model is the absence of the environmental aspect of the disorder, which we cannot mimic in a mouse model. Moreover, the male sex was chosen considering the previous literature on drug addiction models (Martellotta et al., 1998; Mendizábal et al., 2006; Flores et al., 2020). In spite of all these studies previously performed in male rodents, further studies will be necessary to validate these models in female mice and rats.

We have established for the first time a novel and reliable mouse model of cannabinoid addiction using WIN 55,212–2 iv operant self-administration that allows to evaluate three addiction criteria, persistence of response, motivation, and compulsivity, based on the addiction hallmarks defined in the DSM-5. This model also allows to measure two parameters related craving, resistance to extinction and reinstatement, and two phenotypic traits related to cannabinoid addiction, impulsivity and sensitivity to reward. This model represents a pivotal tool to elucidate the neurobiological substrates of cannabinoid addiction and guide future research toward therapeutic strategies to address this disorder.

## Data Availability

The original contributions presented in the study are included in the article/Supplementary Materials, further inquiries can be directed to the corresponding authors.
