# Supplementary figures and images for: A male mouse model of WIN 55,212–2 self-administration to study cannabinoid addiction
Source: Front Pharmacol. 2023 Mar 27;14:1143365. doi: 10.3389/fphar.2023.1143365 (PMC10083303; doi:10.3389/fphar.2023.1143365)

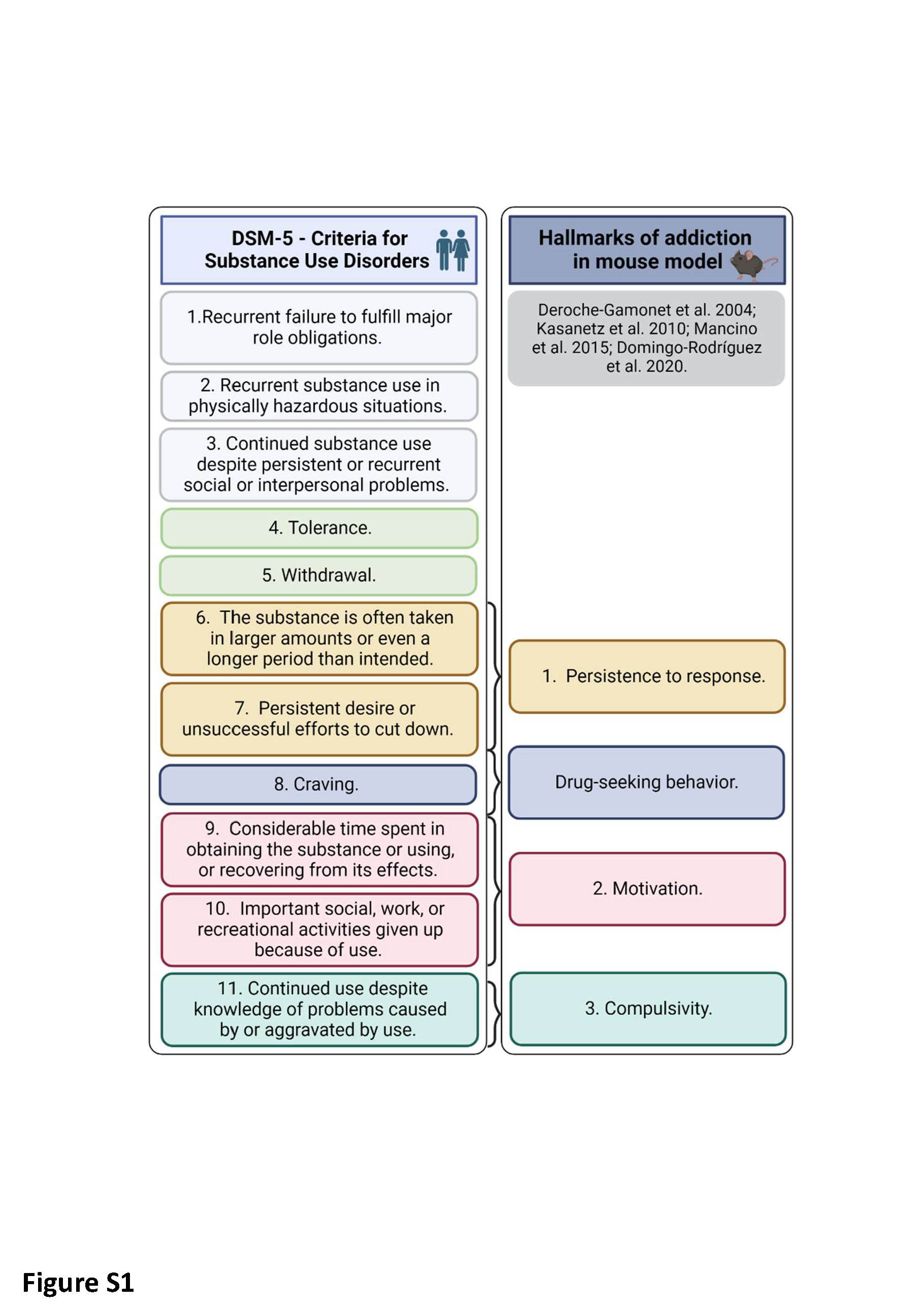

Supplement: Supplementary file 1 [file Image1.JPEG]
