# Supplementary material for: A male mouse model of WIN 55,212–2 self-administration to study cannabinoid addiction
Source: Front Pharmacol. 2023 Mar 27;14:1143365. doi: 10.3389/fphar.2023.1143365 (PMC10083303; doi:10.3389/fphar.2023.1143365)
Supplement: Supplementary file 2 [file DataSheet1.docx]

Supplementary Material

**A male mouse model of WIN 55,212-2 self-administration to study cannabinoid addiction**

**María del Mar Cajiao-Manrique, Elena Martín-García*, Rafael Maldonado***

***Correspondence**: Corresponding Author: [rafael.maldonado@upf.edu](mailto:rafael.maldonado@upf.edu)

[elena.martin@upf.edu](mailto:elena.martin@upf.edu)

**Figure legends**

**SUPPLEMENTARY FIGURE 1.** Comparison between the diagnostic criteria for substance-use disorder of the DSM-5 and their corresponding criteria measured in our mouse model of cannabinoid addiction.

## Supplementary Tables

| **FIGURE 2. WIN 55,212-2 operant self-administration led to the development of an addictive-like phenotype in mice.** | | | | |
| --- | --- | --- | --- | --- |
| **Figure number** | **Statistical analysis** | **Factor name** | **Statistic value** | **P-value** |
| Fig. 2a | Repeated measures ANOVA | FR1 (Sessions 1-5)  Session  Treatment x Session  Treatment  DMS: Actives Saline vs. Inactives Saline  DMS: Actives Saline vs. Inactives CNO  DMS: Actives CNO vs. Inactives Saline  DMS: Actives CNO vs. Inactives CNO  DMS : Actives CNO vs. Actives Saline  DMS : Inactives CNO vs. Inactives Saline | *F*(4, 216)= 0.42  *F*(4, 216)= 1.90  *F*(1, 54)= 27.76 | n.s.  n.s.  p<0.001  p<0.001  p<0.001  p<0.001  p<0.001  n.s.  n.s. |
|  |  | FR2 (Sessions 6-10)  Session  Treatment x Session  Treatment  DMS: Actives Saline vs. Inactives Saline  DMS: Actives Saline vs. Inactives CNO  DMS: Actives CNO vs. Inactives Saline  DMS: Actives CNO vs. Inactives CNO  DMS : Actives CNO vs. Actives Saline  DMS : Inactives CNO vs. Inactives Saline | *F*(4, 216)= 5.98  *F*(4, 216)= 2.54  *F*(1, 54)= 0.01 | p<0.001  p<0.05  p<0.001  p<0.001  p<0.001  p<0.001  p<0.001  n.s.  n.s. |
| Fig. 2  b-d | Kolmogorov-Smirnov | Saline  Persistence  Motivation  Compulsivity  CNO  Persistence  Motivation  Compulsivity | *K-S=* 0.16  *K-S=* 0.38  *K-S=* 0.13  *K-S=* 0.16  *K-S=* 0.25  *K-S=* 0.19 | n.s.  p<0.001  n.s.  n.s.  p<0.05  n.s. |
|  | t.test (equal variances assumed) | Persistence  Motivation  Compulsivity | *t=* 0.56  *U=*85.500  *t=* 0.74 | n.s.  n.s.  n.s. |
|  | Mann-Whitney U |  |  |  |
|  | t.test (equal variances assumed) |  |  |  |
| Fig. 2e | Chi square | Treatment | C-S= 3.77 | n.s. (0.052) |
| Fig. 2  f-h | Kolmogorov-Smirnov | NA Saline  Persistence  Motivation  Compulsivity  A Saline  Persistence  Motivation  Compulsivity  NA CNO  Persistence  Motivation  Compulsivity  A CNO  Persistence  Motivation  Compulsivity | *K-S=*0.16  *K-S=*0.23  *K-S=*0.18  *K-S=*0.36  *K-S=*0.39  *K-S=*0.39  *K-S=*0.17  *K-S=*0.23  *K-S=*0.19  *K-S=*0.26  *-*  *K-S=*0.26 | n.s.  n.s.  n.s.  .  .  .  n.s.  p<0.05  n.s.  .  -  . |
|  | Mann-Whitney U | NA vs A Saline  Persistence  Motivation  Compulsivity  NA vs A CNO  Persistence  Motivation  Compulsivity | *U=*6,500  *U=*6,000  *U=*3,000  *U=*2,000  *U=*6,000  *U=*1,500 | n.s.  n.s.  p<0.05  n.s.  n.s.  p<0.05 |
| Fig. 2  i-k | Pearson correlation | Saline  Persistence and addiction criteria  Motivation and addiction criteria  Compulsivity and addiction criteria  CNO  Persistence and addiction criteria  Motivation and addiction criteria  Compulsivity and addiction criteria | *r=* 0.59  *r=* 0.63  *r=* 0.61  *r=* 0.54  *r=* 0.43  *r=* 0.53 | p<0.05  p<0.05  p<0.05  p<0.05  n.s.  p<0.05 |

| **FIGURE 3. WIN 55,212-2 operant self-administration favored two parameters related with craving and two phenotypic vulnerability traits to addiction-like behavior in mice.** | | | | |
| --- | --- | --- | --- | --- |
| **Figure number** | **Statistical analysis** | **Factor name** | **Statistic value** | **P-value** |
| Fig. 3a | Repeated measures ANOVA | Active lever presses  Session  Treatment x Session  Treatment | *F*(9, 243)= 10.05  *F*(9, 243)= 0.94  *F*(1, 27)= 0.15 | p<0.001  n.s.  n.s. |
|  |  | Inactive lever presses  Session  Treatment x Session  Treatment | *F*(9, 243)= 4.08  *F*(9, 243)= 0.44  *F*(1, 27)= 0.38 | p<0.01  n.s.  n.s. |
| Fig. 3  b-c | Kolmogorov-Smirnov | Saline  Resistance to extinction  Drug-seeking behavior  CNO  Resistance to extinction  Drug-seeking behavior | *K-S=* 0.18  *K-S=* 0.15  *K-S=* 0.26  *K-S=* 0.17 | n.s.  n.s.  n.s.  n.s. |
|  | t.test (equal variances assumed) | Resistance to extinction  Drug-seeking behavior | *t=*2.63  *t=*0.20 | p<0.01  n.s. |
| Fig. 3  d-e | Kolmogorov-Smirnov | NA Saline  Resistance to extinction  Drug-seeking behavior  A Saline  Resistance to extinction  Drug-seeking behavior  NA CNO  Resistance to extinction  Drug-seeking behavior  A CNO  Resistance to extinction  Drug-seeking behavior | *K-S=*0.19  *K-S=*0.20  *K-S=*0.26  *K-S=*0.39  *K-S=*0.27  *K-S=*0.13  *K-S=*0.26  *K-S=*0.26 | n.s.  n.s.  .  .  p<0.05  n.s.  .  . |
|  | Mann-Whitney U | NA vs A Saline  Resistance to extinction  Drug-seeking behavior  NA vs A CNO  Resistance to extinction  Drug-seeking behavior  NA Saline vs NA CNO  Resistance to extinction  Drug-seeking behavior  A Saline vs A CNO  Resistance to extinction  Drug-seeking behavior | *U=*10,500  *U=*8,000  *U=*11,000  *U=*8,000  *U=33*,500  *U=*56,000  *U=*,000  *U=*,000 | n.s.  n.s.  n.s.  n.s.  p<0.05  n.s.  n.s.  n.s. |
| Fig. 3  f-g | Kolmogorov-Smirnov | Saline  Impulsivity  Sensitivity to reward  CNO  Impulsivity  Sensitivity to reward | *K-S=* 0.27  *K-S=* 0.21  *K-S=* 0.22  *K-S=* 0.17 | p<0.05  n.s.  p<0.05  n.s. |
|  | Mann-Whitney U | Impulsivity  Sensitivity to reward | *U=*98,500  *t=*0.39 | n.s.  n.s. |
|  | t.test (equal variances assumed) |  |  |  |
| Fig. 3  h-i | Kolmogorov-Smirnov | NA Saline  Impulsivity  Sensitivity to reward  A Saline  Impulsivity  Sensitivity to reward  NA CNO  Impulsivity  Sensitivity to reward  A CNO  Impulsivity  Sensitivity to reward | *K-S=*0.21  *K-S=*0.18  *K-S=*0.37  *K-S=*0.33  *K-S=*0.22  *K-S=*0.21  *K-S=*0.26  *K-S=*0.26 | n.s.  n.s.  n.s.  .  n.s.  n.s.  n.s.  . |
|  | Mann-Whitney U | NA vs A Saline  Impulsivity  Sensitivity to reward  NA vs A CNO  Impulsivity  Sensitivity to reward  NA Saline vs NA CNO  Impulsivity  Sensitivity to reward  A Saline vs A CNO  Impulsivity  Sensitivity to reward | *U=*11,500  *U=*11,000  *U=*14,000  *U=*6,000  *U=*8,000  *U=*53,500  *U=*11,500  *U=*62,000 | n.s.  n.s.  n.s.  n.s.  n.s.  n.s.  n.s.  n.s. |
| Fig. 3  j-k | Kolmogorov-Smirnov | Saline  BW  Locomotor activity  CNO  BW  Locomotor activity | *K-S=*0.13  *K-S=*0.15  *K-S=*0.17  *K-S=*0.19 | n.s.  n.s.  n.s.  n.s. |
|  | t.test (equal variances assumed) | BW  Locomotor activity | *t=-0.46*  *t=-0.21* | n.s.  n.s. |
